# Changes in lifestyles and depressive symptom among patients with chronic diseases during COVID-19 lockdown

**DOI:** 10.1038/s41598-022-15333-0

**Published:** 2022-07-06

**Authors:** Wei He, Xueyin Zhao, Zhiying Yang, Yan Min, Yi-Hsuan Wu, Qingcong Kang, Eleanor Frost, Peng Gao, Yang Yang, Xinyu Chen, Lijin Chen, Ying Lu, Ann W. Hsing, Shankuan Zhu

**Affiliations:** 1 Chronic Disease Research Institute, The Children’s Hospital, and National Clinical Research Center for Child Health, School of Public Health, School of Medicine, Zhejiang University, Hangzhou, Zhejiang China; 2 Department of Nutrition and Food Hygiene, School of Public Health, Zhejiang University, Hangzhou, Zhejiang China; 3 Stanford Prevention Research Center, Department of Medicine, Stanford University School of Medicine, Stanford, CA USA; 4 Department of Epidemiology and Population Health, Stanford University School of Medicine, Stanford, CA USA; 5 Department of Biomedical Data Science, Stanford University School of Medicine, Stanford, CA USA; 6 Stanford Cancer Institute, Stanford University School of Medicine, Stanford, CA USA

**Keywords:** Diseases, Medical research

## Abstract

This study aims to investigate the impact of COVID-19 lockdown on lifestyle behaviors and depressive symptom among patients with NCDs (noncommunicable diseases). We incorporated a COVID-19 survey to the WELL China cohort, a prospective cohort study with the baseline survey conducted 8–16 months before the COVID-19 outbreak in Hangzhou, China. The COVID-19 survey was carried out to collect information on lifestyle and depressive symptom during lockdown. A total of 3327 participants were included in the COVID-19 survey, including 2098 (63.1%) reported having NCDs at baseline and 1457 (44%) without NCDs. The prevalence of current drinkers decreased from 42.9% before COVID-19 lockdown to 23.7% during lockdown, current smokers from 15.9 to 13.5%, and poor sleepers from 23.9 to 15.3%, while low physical activity increased from 13.4 to 25.2%, among participants with NCDs (P < 0.05 for all comparisons using McNemar's test). Participants with NCDs were more likely than those without to have depressive symptom (OR, 1.30; 95% CI 1.05–1.61), especially among those who need to refill their medication during the COVID-19 lockdown (OR, 1.52; 95% CI 1.15–2.02). Our findings provide insight into the development of targeted interventions to better prepare patients with NCDs and healthcare system to meet the challenge of future pandemic and lockdown.

## Introduction

Since 2020, Coronavirus Disease 2019 (COVID-19) has been diagnosed in over 500 million individuals and caused over six million deaths worldwide as of May 4th, 2022^1^, posing an unprecedented challenge to the healthcare system. In China, a total of 1,210,601 cases have been confirmed, including 15,372 deaths^1^. Between February and March 2020, China issued a two-month lockdown due to COVID-19 pandemic, mandating all individuals (unless authorized otherwise) to stay at home and stop outdoor activity and social gathering^2^. During the lockdown period, people’s lives changed drastically, and depressive symptom is likely to be triggered^3–5^.

COVID-19 patients who died were generally older, with a history of noncommunicable diseases (NCDs)^6,7^. Therefore, patients with NCDs may experience more psychological burden during the COVID-19 pandemic or COVID-19 lockdown. It is estimated that 244.5 million people have been diagnosed with hypertension^8^, 92.4 million with diabetes^9^, and 94 million with cardiovascular disease in China^10^. These patients generally have a much higher risk of COVID-related morbidity and mortality, and even without COVID infection, their treatment for NCD and quality of life can be affected during the pandemic lockdown due to the impact of health care access and medication refill. Few studies have investigated the impact of COVID-19 lockdown on NCD patients in a community setting.

Within the WELL China cohort that has collected baseline data 8–16 months before COVID-19, we incorporated a survey one month after the COVID-19 lockdown to investigate the impact of COVID-19 lockdown on lifestyle behaviors and depressive symptom among 3327 cohort members in the Gongshu District, Hangzhou, China.

## Results

### Baseline characteristics

Table 1 lists the baseline characteristics of 3327 study participants by NCDs status. Over three in five (63.1%) of the participants had NCDs. As shown, participants with NCDs were more likely to be older, widowed, obese, and having worse sleep quality.

**Table 1:** Baseline characteristics among 3327 participants with and without non-communicable diseases.

Baseline characteristics	Non-communicable diseases*		*P* value*
	No (n = 1229)	Yes (n = 2098)	
**Age (years)**			< 0.001
< 45	377 (30.7)	257 (12.2)	
45–64	688 (56.0)	1114 (53.1)	
≥ 65	164 (13.3)	727 (34.7)	
**Gender**			0.912
Male	461 (37.5)	791 (37.7)	
Female	768 (62.5)	1307 (62.3)	
**Marital status**			< 0.001
Unmarried	86 (7.0)	58 (2.8)	
Married	1080 (88.0)	1882 (89.7)	
Divorced	31 (2.5)	57 (2.7)	
Widowed	30 (2.4)	101 (4.8)	
Unknown	2	0	
**Body mass index (kg/m**^**2**^**)**			< 0.001
< 25	906 (73.8)	1309 (62.4)	
25–29	291 (23.7)	691 (33.0)	
≥ 30	30 (2.4)	97 (4.6)	
Unknown	2	1	
**Smoking**			< 0.001
Never	938 (76.5)	1621 (77.3)	
Former	48 (3.9)	144 (6.9)	
Current	240 (19.6)	333 (15.9)	
Unknown	3	0	
**Drinking**			0.005
Never	722 (58.9)	1157 (55.1)	
Former	10 (0.8)	43 (2.0)	
Current	494 (40.3)	898 (42.8)	
Unknown	3	0	
**Eating irregularly**			0.040
No	1153 (94.5)	2014 (96.0)	
Yes	67 (5.5)	83 (4.0)	
Unknown	9	1	
**Sleep quality**^**†**^			< 0.001
Very good	331 (27.1)	46 6(22.2)	
Fairly good	681 (55.7)	1132 (54.0)	
Fairly bad	190 (15.5)	443 (21.1)	
Very bad	20 (1.6)	56 (2.7)	
**Physical activity**			< 0.001
Low	235 (20.9)	281 (14.0)	
Moderate	601 (53.4)	1181 (58.9)	
Vigorous	289 (25.7)	544 (27.1)	
Unknown	104	92	

Data were presented as number (%).

*p value was derived from Chi-Square test.

^†^Sleep quality was self-reported as very good, fairly good, fairly bad, or very bad.

### Lifestyle changes during COVID-19 lockdown

**Figure 1:**
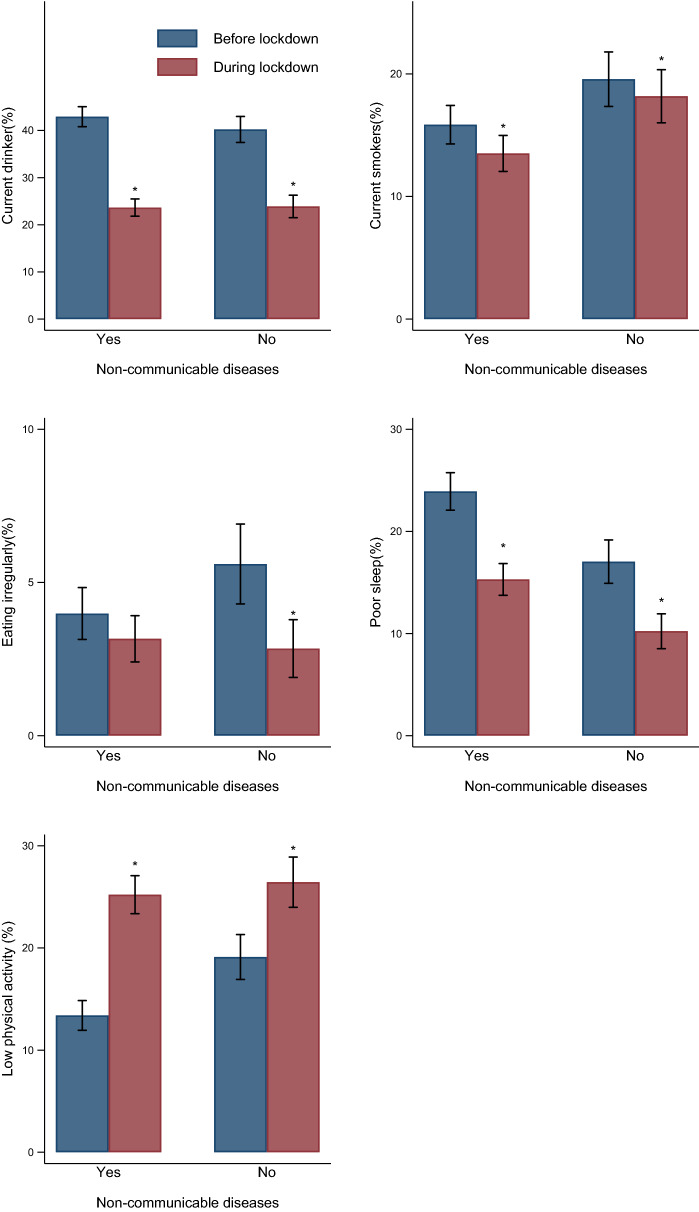
Lifestyle before and during the nationwide COVID-19 lockdown in Hangzhou, China. *p < 0.05, for the difference before and during lockdown using McNemar's test.

Figure 1 shows lifestyle changes among study participants by NCD status. Among individuals with NCDs, the prevalence for current alcohol drinkers and current smoker decreased from 42.9 to 23.7% (P < 0.05 using McNemar's test) and 15.9–13.5% (P < 0.05 using McNemar's test), respectively. Participants with self-reported poor sleep quality decreased from 23.9 to 15.3% (P < 0.05 using McNemar’s test). However, low physical activity increased from 13.4 to 25.2% (P < 0.01 using McNemar's test). Similar patterns were found when we restricted our analyses to participants without NCDs.

### Changes in prevalence of depressive symptom during COVID-19 lockdown

**Figure 2:**
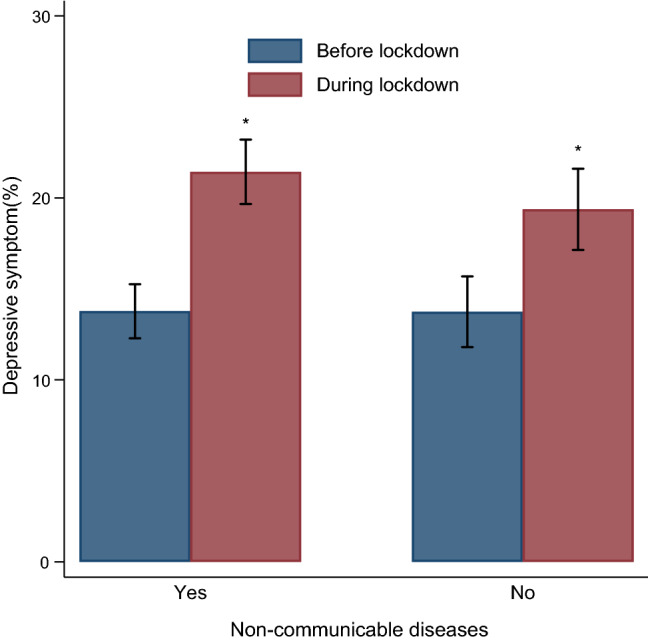
Depressive symptom before and during the nationwide COVID-19 lockdown in Hangzhou, China. *p < 0.05, for the difference before and during lockdown using McNemar's test.

Figure 2 shows the prevalence of depressive symptom among individuals with and without NCDs before COVID and during the COVID-19 lockdown. Among individuals with NCDs, the prevalence of depressive symptom during COVID-19 lockdown was 21.4% (95 CI 19.6–23.2%), significantly higher than the 13.8% (95% CI 12.3–15.3%) observed at baseline before the COVID-19 lockdown. Similar results were seen for individuals without NCDs.

Table 2 shows the associations between selected characteristics and incident depressive symptom during the lockdown. As shown, younger age and the need to refill medication were associated with a higher risk of having incident depressive symptom during COVID-19 lockdown, whereas maintaining moderate to vigorous physical activity was associated with lower risk of depressive symptom. Further analyses combining NCDs and need to refill medication found that, as compared with no NCDs, NCDs with no need to refill medications was associated with 1.29 (95% Cis 1.04–1.58) times increased risk, and NCDs with need to refill medication was associated with 1.48 (95% CI 1.07–2.06) times increased risk of having incident depressive symptom during COVID-19 lockdown.

**Table 2:** Selected characteristics and their relation with incident depressive symptom during the COVID-19 lockdown (n = 2820).

	Incident depressive symptom during lockdown No./total (%)s	N	Odds ratios (95% CIs)
		Age-adjusted*	Multivariable adjusted^†^
**Baseline characteristics**			
Age (years)			
< 45	126/499 (25.3)	1.60 (1.26–2.04)	1.69 (1.31–2.17)
45–64	271/1554 (17.4)	1.00 (reference)	1.00 (reference)
≥ 65	149/767 (19.4)	1.14 (0.91–1.42)	1.06 (0.85–1.34)
Non-communicable disease			
No	183/1042 (17.6)	1.00 (reference)	1.00 (reference)
Yes	363/1778 (20.4)	1.31 (1.07–1.62)	1.30 (1.05–1.61)
Gender			
Male	214/1066 (20.1)	1.00 (reference)	–
Female	332/1754 (18.9)	0.94 (0.78–1.14)	–
Marital status			
Unmarried	23/98 (23.5)	0.96 (0.58–1.59)	–
Married	484/2549 (19.0)	1.00 (reference)	–
Divorced	12/66 (18.2)	0.93 (0.49–1.75)	–
Widowed	27/107 (25.2)	1.50 (0.95–2.37)	–
Body mass index, kg/m^2^			
< 25	352/1861 (18.9)	1.00 (reference)	–
25–29	169/852 (19.8)	1.09 (0.89–1.34)	–
≥ 30	24/104 (23.1)	1.26 (0.79–2.02)	–
Unknown	1/3	–	–
Smoking			
Never	419/2162 (19.4)	1.00 (reference)	–
Former	33/174 (19.0)	1.01 (0.68–1.49)	–
Current	94/483 (19.5)	1.01 (0.79–1.29)	–
Unknown	0/1	–	–
Drinking			
Never	323/1585 (20.4)	1.00 (reference)	1.00 (reference)
Former	14/44 (31.8)	1.91 (1.00–3.65)	1.84 (0.95–3.55)
Current	209/1190 (17.6)	0.83 (0.68–1.00)	0.84 (0.69–1.02)
Unknown	0/1	–	–
Eating irregularly			
No	525/2710 (19.4)	1.00 (reference)	–
Yes	20/105 (19.0)	0.92 (0.56–1.52)	–
Unknown	1/5	–	–
Sleep quality			
Very good	142/707 (20.1)	1.00 (reference)	–
Fairly good	302/1615 (18.7)	0.89 (0.71–1.11)	–
Fairly bad	95/454 (20.9)	1.03 (0.77–1.38)	–
Very bad	7/39 (17.9)	0.89 (0.39–2.07)	–
Physical activity			
Low	82/406 (20.2)	1.00 (reference)	–
Moderate	272/1524 (17.8)	0.91 (0.69–1.20)	–
Vigorous	161/739 (21.8)	1.19 (0.88–1.61)	–
Unknown	31/151	–	–
**Characteristics during lockdown**			
Maintaining moderate to vigorous physical activity			
No	197/850 (23.2)	1.00 (reference)	1.00 (reference)
Yes	268/1494 (17.9)	0.76 (0.62–0.94)	0.77 (0.62–0.95)
Unknown	81/476	–	–
Need to refill medication			
No	343/2051 (16.7)	1.00 (reference)	1.00 (reference)
Yes	83/352 (23.6)	1.62 (1.23–2.15)	1.52 (1.15–2.02)
Unknown	120/417	–	–

Dashes indicate inapplicable or not included in the model. Women with depressive symptom at baseline were excluded from this analyses.

*Variables were not included in the multivariable model if they were not significantly associated with incident depressive symptom in the univariable models.

^†^Adjusting all variables listed in this column.

## Discussion

In this large population-based survey, we showed that during the COVID-19 lockdown in China, the prevalence of low physical activity and depressive symptom increased, while the prevalence of current smoking, drinking, and poor sleep quality decreased. Younger age (< 45 years) and the need for medication refill were associated with a higher risk of having incident depressive symptom during the lockdown, whereas maintaining moderate to vigorous physical activity was associated with lower risk of having depressive symptom during COVID-19 lockdown.

Our finding on lifestyle behavior changes during the lockdown period in China provides information on the impact of the COVID-19 pandemic. The lockdown prohibited people from going out and exercising outdoors, likely resulting in the higher prevalence of low physical activity and excessive sitting, which may translate to increased risk of chronic diseases^11^. In contrast, reduced alcohol drinking and smoking, and improved sleep quality are positive changes related to health and well-being during this lockdown period. It is possible that NCDs patients may benefit from the lockdown by reducing risky behaviors and improved sleep. Follow up and further research should investigate the short-term benefits of these behavior changes in patients with NCDs and whether these improvements in healthy lifestyle remain after the lockdown period.

Depressive symptom increased from 13.7% at baseline (before lockdown) to 20.7% during lockdown, suggesting an adverse impact of lockdown on mental health. This results in consistent with previous findings on general population^12^. Given the extent of the COVID-19 lockdown worldwide, this high prevalence of depressive symptom suggests that millions of individuals may experience mental health stress during the COVID-19 lockdown. Traditional psychological crisis intervention, e.g., face-to-face contact with qualified mental health professionals, is not likely to be readily available to all who need it. Psychological service system should thus be, and is being re-formed to incorporate novel strategies (e.g. online mental health service and telephone-based psychological counselling) to better tackle psychological problem during the COVID-19 epidemic^13,14^.

Lack of medical support may also lead to newly emerged depressive symptom among NCDs patients since they were not able to visit the clinic in person for follow up, in particular for imaging or examinations that need in-person service. In addition, in our study, the need to refill medication was associated with a higher risk of having incident depressive symptom during COVID-19 lockdown, suggesting that lack of medication for their chronic conditions may result in concerns and anxiety, leading to depressive symptoms. This concern is further highlighted by the fact that over 70% of patients with chronic diseases have reported inadequate availability of medicine during COVID-19 lockdown^15^. Collectively, these data suggest the importance of providing adequate clinical support, either through telemedicine or special arrangements (e.g., involving community pharmacists^16^ or delegating someone else for medication pick-up^17^), for high-risk individuals, such as patients with chronic diseases during future outbreaks or lockdown to minimize mortality. In addition, mental health of patients with chronic disease needs to be taken into account to minimize anxiety and mental breakdown to minimize the deterioration of the underlying conditions.

The key strength of our study is having longitudinal data on the same individuals before and during COVD-19 in a large population-based study. Limitation of the study should be noted. First, all our data are self-reported, thereby some misclassification is possible. Second, the follow up survey was carried out one month after the lockdown already completed, thereby some recall issues may contribute to misclassification of information. Third, generalizability of our results is limited as access to health care and support vary greatly in different country.

In conclusion, in this community-based study, we found that COVID-19 lockdown significant impact lifestyle and quality of life among individuals with chronic diseases. These results provide the basis for targeted prevention to better prepare NCD patients, community, and healthcare system to meet the challenge in future outbreaks or lockdown.

## Methods

### Study population

Subjects were a subset of the Wellness Living Laboratory (WELL)-China study. Details of the WELL-China cohort are Described elsewhere^18–20^. Briefly, WELL-China is a population-based cohort for the investigation of well-being in Hangzhou, Zhejiang province, China. In this COVID-19 Study, we surveyed the 4144 WELL-China participants who completed their baseline survey in Gongshu District, Hangzhou, China, between October 2018 and May 2019, which is 8–16 months before the COVID-19-related nationwide lockdown.

For the COVID-19 survey, we conducted a telephone survey in April 2020, around one month after the lockdown, to collect information on lifestyle, mental health and medical access during the 2-month lockdown (between February and March, 2020; Shelter in Place) period. Of the 4144 participants at baseline, 3356 responded to the telephone survey (81% response rate). We excluded 27 participants who did not report their baseline NCDs conditions, leaving 3329 for the final analysis.

The WELL-China project was approved by the ethics review board at both Zhejiang University (No. ZGL201507-3) in China and Stanford University (IRB-35020) in USA. All participants provided written informed consent. The follow-up telephone interview was further approved by the ethics review board at Zhejiang University (No. ZGL202004-01). We conducted this study in accordance with the latest revised ethical guidelines of the Declaration of Helsinki.

### Baseline data collection

During 2018–2019, face-to-face interviews were performed to collect baseline characteristics before the COVID-19 outbreak. Information on noncommunicable diseases (NCDs) was assessed and included self-reported history of diagnosed diabetes, hypertension, cardiovascular disease, cancer, endocrine and metabolic diseases, osteoarthritis, digestive system diseases, respiratory system diseases, mental diseases, urinary system diseases, nervous system diseases, immune diseases, and allergies at baseline. Smoking status and alcohol consumption were categorized as never, former, or current. Sleep quality was self-reported as very good, fairly good, fairly bad, or very bad. Eating behavior was categorized into not regular, mostly regular, or very regular. Physical activity was measured using the short version of the International Physical Activity Questionnaire, and were classified into three categories: low; moderate; and vigorous^21^.

### Follow-up data collection

We used telephone follow-up survey to collect the same information on lifestyles and WHO-5 as well as medication refill during the lockdown period. Participants who reported moderate to vigorous physical activity both at baseline and during follow-up survey was defined as maintaining moderate to vigorous physical activity.

### Depressive symptom

The WHO-Five Well-being Index (WHO-5) was used to assess depressive symptom. The WHO-5 tool is a five-item self-report instrument that assesses well-being (e.g., “I have felt calm and relaxed”) over the past two weeks on a 6-point Likert scale (0 = “not present,” to 5 = “constantly present”). Scores on the WHO-5 range from 0 to 25, and higher scores indicate greater well-being. The WHO-5 has demonstrated good reliability and validity and the ability to identify adults experiencing depression in medical settings in several countries across Africa, Asia, Australia, Europe, North America, and South America^22^.

The five items included: (1) “I have felt calm and relaxed”, (2) “I have felt cheerful and in good spirits”, (3) “I have felt active and vigorous”, (4) “My daily life has been filled with things that interest me” and (5) “I woke up feeling fresh and rested”^22^. Depressive symptom was then defined as the participants has answered 0 (at no time) to 1 (some of the time) to any of the five items or if the WHO-5 raw score below 13^23^.

Participants who were classified to have depressive symptom in 2020 during the COVID-19 lockdown but not at baseline (2018–2019) were classified as having incident depressive symptom.

### Statistical analysis

Chi-square tests were used to assess whether baseline characteristics differed between participants with and without NCDs. McNemar's tests were used to investigate the change in lifestyle and mental health between before and during the COVID19 lockdown.

Univariable and multivariable logistic regressions were used to investigate the association between baseline characteristics and incident depressive symptom during COVID-19 lockdown, among participants with and without NCDs.

We used STATA, version 13 (STATA, College Station, TX), for all the analyses. All statistical tests were two-sided, and statistical significance was defined as P < 0.05.

## Data Availability

The datasets generated and analyzed during the current study are not publicly available due to protection of participant privacy and confidentiality but are available from the corresponding author on reasonable request.
